# BLURRING BOUNDARIES: QUALITATIVE RESEARCH ON DIRECT CARE WORKERS’ EXPERIENCES

**DOI:** 10.1093/geroni/igae098.3387

**Published:** 2024-12-31

**Authors:** Brittney Pond

**Affiliations:** University of California San Francisco, San Francisco, California, United States

## Abstract

In the United States, there are 4.8 million direct care workers who provide care and services to older adults and people living with disabilities (PHI, 2023). Research suggests that direct care workers in home and community-based services occupy a unique role between family and non-family. In this poster, I discuss how direct care workers’ close relationships with their clients often led to blurring personal and professional boundaries that created challenges through semi-structured interviews with 43 (n=43) direct care workers providing care and services to older adults in the U.S. This research addresses two primary research questions: 1) what is the nature of work and relationships with clients for paid direct care workers? 2) how do direct care workers for non-family older adults navigate personal and professional boundaries? Through these interviews, I found that direct care workers unique roles within home care settings often caused challenges such as doing or feeling pressure to do additional labor on and off their shifts and hesitancy to be away from their clients. These findings support and expand upon existing literature that direct care workers often form close, reciprocal relationships with non-family clients and often participate in additional labor beyond their work requirements.

